# Inhibition of Tunneling Nanotubes between Cancer Cell and the Endothelium Alters the Metastatic Phenotype

**DOI:** 10.3390/ijms22116161

**Published:** 2021-06-07

**Authors:** Chinmayee Dash, Tanmoy Saha, Shiladitya Sengupta, Hae Lin Jang

**Affiliations:** 1 Center for Engineered Therapeutics, Department of Medicine, Brigham and Women’s Hospital, Harvard Medical School, Boston, MA 02139, USA; cdash@bwh.harvard.edu (C.D.); tsaha2@bwh.harvard.edu (T.S.); 2 Division of Health Science and Technology, Harvard-Massachusetts Institute of Technology, Massachusetts Institute of Technology, Boston, MA 02139, USA; 3 Dana Farber Cancer Institute, Boston, MA 02215, USA

**Keywords:** tunneling nanotube (TNT), metastasis, RhoGTPase inhibitor, phenotypic plasticity, exocyst complex, actin remodeling, 3D culture, mammosphere, cellular aggregates

## Abstract

The interaction of tumor cells with blood vessels is one of the key steps during cancer metastasis. Metastatic cancer cells exhibit phenotypic state changes during this interaction: (1) they form tunneling nanotubes (TNTs) with endothelial cells, which act as a conduit for intercellular communication; and (2) metastatic cancer cells change in order to acquire an elongated phenotype, instead of the classical cellular aggregates or mammosphere-like structures, which it forms in three-dimensional cultures. Here, we demonstrate mechanistically that a siRNA-based knockdown of the exocyst complex protein Sec3 inhibits TNT formation. Furthermore, a set of pharmacological inhibitors for Rho GTPase–exocyst complex-mediated cytoskeletal remodeling is introduced, which inhibits TNT formation, and induces the reversal of the more invasive phenotype of cancer cell (spindle-like) into a less invasive phenotype (cellular aggregates or mammosphere). Our results offer mechanistic insights into this nanoscale communication and shift of phenotypic state during cancer–endothelial interactions.

## 1. Introduction

Metastasis is one of the foremost causes of cancer-related mortality [1]. It involves a cascade of events, including break out of cancer cells from the primary tumor location, invasion and intravasation through the blood vessels, and colonization at the secondary site in the patient’s body [2,3]. To invade the blood vessel barrier, which is lined with endothelial cells, tumor cells communicate with the endothelial cells and impose phenotypical transition [4,5,6,7,8,9] to enter the circulation and achieve metastasis [10,11,12]. Cross talk between the cancer cells and the endothelial cells takes place through various cellular communications, such as paracrine signaling, through physical modalities like gap junctions, synapses, exosomes, and vesicles that contribute to cancer progress and metastasis [13,14,15,16].

Tunneling nanotubes (TNTs) are one such means of physical communication and allows direct cell-to-cell communication over larger distances, forming huge networks that connect many cells [17]. TNTs are actin-based membrane extensions that perform a significant role in the long-distance transfer of cytoplasmic contents, various plasma membrane proteins, cell organelles, ions, and metabolites [18,19]. TNTs exhibit high variability in size, ranging from about 50 nm to up to around 1 µm in thickness, with an average length ranging approximately from 30 µm to 200 µm [7,17,19,20]. TNT communications between cells were first described in cultured rat pheochromocytoma PC12 cells [17] and were later identified in numerous cell types [21,22,23,24]. In our previous study, we reported that the TNTs acted as physical conduits for the transfer of microRNAs from cancer cells to endothelial cells, upregulating the markers associated with pathological endothelium [7]. Fluorescence microscopy revealed the presence of F-actin in the nanotubular structures [7,17,20,25]. However, the process involved in the formation of these TNTs remains unclear. Two major mechanism models—actin-driven protrusion and cell-dislodgement mechanisms—have widely been used to understand the formation of these nanotubes, implying either that the cells extend their filopodia-like structures, or that two cells merge their cellular membranes to interact with each other at a distance [25]. To distinguish TNTs from other cellular membrane structures, TNTs are often additionally defined as long nanotubular structures that hover above the substratum, unlike filopodia [7,25]. The reduction of TNT formation using F-actin depolymerizing compounds, such as Latrunculin and Cytochalasin D, has been reported in the case of both of the model systems—actin-driven protrusion and cell-dislodgement mechanism—that mediate the formation of TNTs [7,17,26]. This suggests that actin polymerization acts as an integral part in the process of TNT formation [27,28]. However, the actual mechanism underlying TNT formation between the endothelium and the cancer cells has not been well studied.

We hypothesized that TNTs formed between the cancer cells and the endothelium were mediated by the actin polymerization pathway, emphasizing the involvement of Rho GTPase family of proteins along with the exocyst complex [29,30,31]. We observed that the proteins associated with actin polymerization pathway colocalize with the TNT formed between the cancer cells and the endothelium. Furthermore, pharmacological inhibitors of Rho GTPase reduced the number of TNT formation together with the transfer of cytoplasmic contents between the cancer cells and the endothelium. Additionally, in the present study, we observed the phenotypic transition of the cancer cells from less invasive 3D cellular aggregates or mammosphere-like structures to highly invasive spindle-shaped structures in the presence of the endothelial cells. We observed a reversal of this phenotypic transition of cancer cells and endothelial cells in the presence of inhibitors of TNT formation.

## 2. Results

### 2.1. Cancer Cells Acquire an Invasive Phenotype by Interacting with the Endothelium

**Figure 1:**
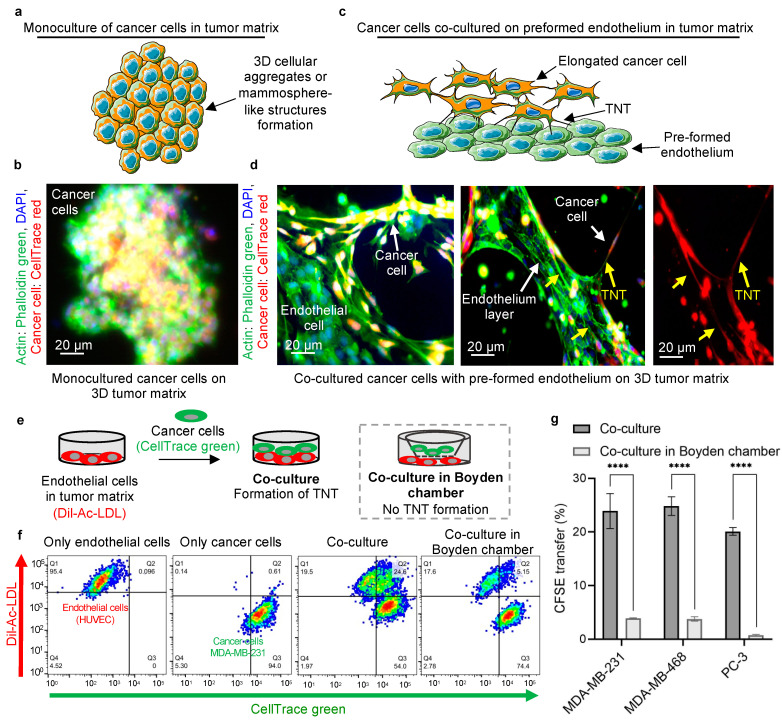
Communication of cancer cells with endothelium and phenotypic transition. (**a**) Schematic representation of the monoculture of metastatic cancer cells (MDA-MB-231) in the 3D tumor matrix, forming 3D cellular aggregates or mammosphere-like structures. (**b**) Schematic for cancer cells adopting an elongated phenotype when co-cultured with preformed endothelium in the 3D tumor matrix. The elongated phenotype of cancer cells aligns with the endothelial layer, forming nanoscale communications with the endothelial cells. (**c**) Representative image of the formation of mammospheres during the monoculture of cancer cells (MDA-MB-231) in the 3D tumor matrix in the absence of endothelium. MDA-MB-231 cancer cells were stained with CellTrace red and cultured on a Matrigel (tumor matrix) coated plate for 24 h. The cells were fixed, actin filaments stained with phalloidin green, and the nuclei were counterstained with DAPI. The yellow signal from the image originates from the overlapping of CellTrace red stained cancer cell and phalloidin green staining. (**d**) Representative image of the co-culture of cancer cells (MDA-MB-231) with preformed endothelium of human umbilical vein endothelial cells (HUVECs) for 24 h in the 3D tumor matrix. HUVECs (unstained) were cultured on Matrigel-coated plate for 5–6 h to form the endothelial network. After that, CellTrace red stained cancer cells were added to the preformed endothelium. After 24 h of co-culture, the cells were fixed, and actin filaments were stained with phalloidin green, and the nuclei were counterstained with DAPI. The yellow and green stained cells represent the cancer cells (CellTrace red and phalloidin green stained) and endothelial cells (phalloidin green stained), respectively. The image on the left side reveals the cancer cells (yellow) have transformed into an elongated phenotype and aligned with the preformed endothelial layer (green). The right-side image shows the physical communication between the cancer cells and the endothelial cells via tunneling nanotubes (yellow arrows). The phalloidin staining signifies the presence of actin in the nanotubes. The monochromatic (red) image represents the cancer cells (red fluorescence) obtaining an elongated phenotype, as compared with the mammosphere-like structure in the monoculture of cancer cells in the 3D tumor matrix, by forming nanotubular structures with the endothelium and invaginating into the endothelial network. (**e**) Schematic representation of the experimental design to evaluate the transfer of cytoplasmic contents from cancer cells to endothelial cells. MDA-MB-231 cancer cells and HUVECs were loaded with CellTrace green (CFSE) and Dil-Ac-LDL, respectively, and employed in the co-culture setup for 24 h in the 3D tumor matrix. The transfer of CFSE-labeled cytoplasmic components from cancer cells to endothelial cells was evaluated. The co-culture was also compared in a controlled setup in a Boyden chamber, where the cancer cells and endothelial cells were added to the upper and lower chambers, respectively, separated by a membrane with a pore size of 0.4 µm. Hence, the direct nanoscale communication between cells was forbidden, whereas the exosomal and paracrine communications was allowed. (**f**) The representative FACS plot for monoculture of each cell type, HUVECs (red) and MDA-MB-231 cancer cells (green), in the respective fluorescence channel, wherein the co-culture for cancer cells and endothelial cells shows a CFSE transfer to the endothelial cells of 24.6%, while there is 5.15% CFSE transfer to endothelial cells for the co-culture in the Boyden chamber. (**g**) Graph showing quantitative CFSE transfer from three different highly metastatic cancer cell lines (MDA-MB-231, MDA-MB-468 and PC-3 cells) to endothelial cells in the case of direct co-culture and co-culture in a Boyden chamber. A significantly high amount of CFSE transfer was observed in case of direct co-culture. Data represent mean ±SEM of three experiment (*n* = 3), and statistical analysis was performed with two-way ANOVA following Sidak’s multiple comparison test, **** *p* < 0.0001.

The tumor microenvironment can affect the phenotypic plasticity of the metastatic cancer cells. Indeed, monocultures of MDA-MB-231 cancer cells on the three-dimensional (3D) tumor matrix acquire a typical mammosphere-like structure (Figure 1a,b and Supplementary Figure S1a,b). In contrast, we observed an invasive, elongated phenotype of cancer cells when they were added to the preformed endothelium on the 3D tumor matrix (Figure 1c,d). This elongated phenotype has been reported to be associated with an invasive potential [32]. Consistent with our previous report [7], thin nanoscale bridges were found between cancer cells and the endothelial cells (Figure 1d, Supplementary Figure S1c,d). We have previously shown that such a spindle shaped morphology facilitates the migration of cancer cells though the endothelial barrier [7,33,34]. As shown in Figure 1d, the nanotubular structures were composed of actin filaments, revealed by phalloidin staining in those TNTs. Similar to previous reports, we also observed varied lengths of nanotubular structures between the cancer cells and the endothelial cells (Figure 1d) [7,17,33]. To further strengthen our hypothesis, we tested the phenotypic plasticity in another highly metastatic human breast cancer cell line, MDA-MB-468, and a highly metastatic human prostate cancer cell line, PC-3. In monoculture of cancer cells, we observed mammosphere formation in the case of MDA-MB-468 cancer cells and similar 3D cellular aggregates of PC-3 cancer cells in the 3D tumor matrix (Supplementary Figure S1a,b). However, in the co-culture of MDA-MB-468 or PC-3 cancer cells with the endothelial network in the 3D tumor matrix, an elongated phenotype of the cancer cells was observed. Nanotubular communication of cancer cells (MDA-MB-468 or PC-3 cells) with endothelial cells was also observed, which was in alignment with the results obtained for MDA-MB-231 cells (Supplementary Figure S1c,d). On the other hand, we used a poorly metastatic MCF-7 breast cancer cell line as a negative control. No TNTs were found between MCF-7 cancer cells and endothelial cells in the tumor matrix, and the formation of mammosphere-like structures by MCF-7 cancer cells was also observed in the co-culture with endothelial cells. In the absence of TNT communication between the cancer cells and the endothelial cells, MCF-7 cancer cells were found to form mammospheres even in the presence of endothelium in the co-culture setup, probably because of their less invasive phenotype (Supplementary Figure S1e).

Trafficking of cytoplasmic components and cell organelles through nanotubes has been well documented in the literature [24,26,35,36]. In this study, we firstly investigated the transfer of cytoplasmic components through the nanobridges formed between different metastatic cancer cell lines with the endothelial cells. To quantify this intercellular transfer, the cancer cells were stained with CellTrace green (carboxyfluorescein succinimidyl ester or CFSE, which preferentially binds to intracellular lysine residues and other amine sources) before adding them to the co-culture of Dil-Ac-LDL-labeled endothelial cells (endothelial cells were labeled with tdTomato in the case of co-culture with MDA-MB-468 and PC-3 cancer cells). The cells were co-cultured in a 3D tumor matrix and analyzed by FACS after 24 h (experimental schematic, Figure 1e). We observed the transfer of CFSE from MDA-MB-231 cancer cells to the endothelial cell (Dil-Ac-LDL +ve) population, resulting in an endothelial population positive for both Dil-Ac-LDL and CellTrace green or CFSE (double positive red and green population) (a representative gating strategy is shown in Supplementary Figure S2) [7,24,36]. Similar double positive populations of endothelial cells (both tdTomato and CFSE +ve) were also observed in the case of MDA-MB-468 and PC-3 cancer cells (Supplementary Figure S3b,c with a representative gating strategy being shown in Supplementary Figure S3d). Almost 20–25% of the endothelial population was found to have accepted CFSE-labeled cytoplasmic components from the cancer cells (Figure 1f and Supplementary Figures S2 and S3). As a negative control, the co-culture was set up in a Boyden chamber, where two chambers are separated by a membrane with a pore size of 0.4 µm (Figure 1e,f).

We cultured cancer cells and endothelial cells in the upper and lower compartments of a Boyden chamber, respectively, which allows the exosomal and paracrine communication between the cells but restricts physical communication [37]. A significantly reduced transfer of cytoplasmic components was observed in case of Boyden chamber assay (Figure 1f). Only ~0.5–5% of endothelial cells were found to have accepted CFSE from cancer cells (Figure 1g and Supplementary Figure S3) which were consistent with the previous reports for exosome-mediated transfer [21].

In Figure 1f, there is a difference in cancer cell population in the case of both the direct co-culture setup (Q3 54%) and the co-culture setup in the Boyden chamber (Q3 74%). The difference is mainly because of the difficulty in collecting the cells from the Matrigel (tumor matrix) layer, leading to a change in the relative population of cells. We usually lose a portion of the overall population while recovering the cells from the Matrigel in order to prepare the single-cell suspension for the flow cytometric analysis. In the case of the direct co-culture setup, both the cancer cells and the endothelial cells were co-cultured in the tumor matrix. As both the cells were collected from Matrigel, the loss of each cell type is equal, resulting in the relative population of cancer cells and endothelial cells being the same. On the other hand, in the case of the co-culture setup in the Boyden chamber, the endothelial cells were collected from the lower chamber (with Matrigel), whereas the cancer cells were collected from the upper chamber of the Boyden chamber (without Matrigel). Hence, the loss of cancer cells remains negligible, whereas the loss of endothelial cells recovered from the Matrigel remains same in case of co-culture in Boyden chamber. As a result, FACS plots showed different relative populations (%) of endothelial cells between the direct co-culture system and the co-culture system using the Boyden chamber, even though the absolute numbers of endothelial cells were similar. In other words, the difference observed in relative populations of endothelial cells between two co-culture systems is because of relative change in the cancer cell population.

To overcome the possibility of CFSE dye leakage from the CFSE stained cancer cells and non-specifically stain the endothelial cells in the co-culture setup, a conditioned media experiment was performed. The cancer cells (MDA-MB-231 cells) were stained with CFSE and then cultured in complete media. After 24 h, the conditioned media from the cancer cells were added to HUVECs (tdTomato labeled) in the 3D tumor matrix. Simultaneously, the cancer cells stained with CFSE were added to a separate set of tdTomato-labeled HUVECs in the tumor matrix following a co-culture setup (Supplementary Figure S4a). No significant CFSE staining of HUVECs was observed when they were cultured with the conditioned media of cancer cells in the tumor matrix. Only 0.15% of endothelial cells were found to have both tdTomato +ve and CFSE +ve. Whereas a considerable amount of CFSE transfer was observed when the CFSE stained cancer cells were added to the tdTomato-labeled endothelial cells in the tumor matrix, which resulted in 25% of tdTomato +ve and CFSE +ve endothelial cells (Supplementary Figure S4b). This additional experiment confirmed the absence of CFSE leakage from the cancer cells in the co-culture assay.

### 2.2. Mechanism of Nanoscale Bridge Formation and Involvement of Small GTPase–Exocyst Complex

**Figure 2:**
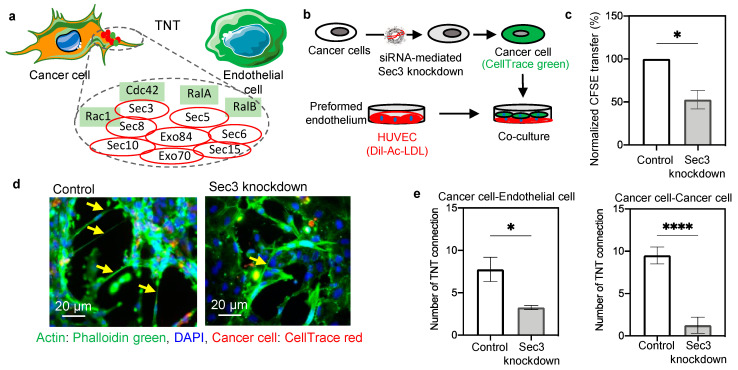
Involvement of exocyst proteins in TNT formation. (**a**) Schematic representation of the nanotube connection between a cancer cell and an endothelial cell and the involvement of actin remodeling pathway. The involvement of the exocyst complex and small GTPases in actin polymerization and TNT formation is shown schematically. The exocyst complex, is a combination of eight proteins (Sec3, Sec5, Sec6, Sec8, Sec10, Sec15, Exo70, and Exo84) that interact with small Ras GTPases (like, Cdc42, Rac1, RalA/B) to fulfill the energy requirement for the actin polymerization. (**b**) Schematic representation of the experimental design of the Sec3 knockdown in the cancer cells and the co-culture setup with preformed endothelium. (**c**) Graph showing the reduction of CFSE transfer upon knockdown of Sec3 (one of the major exocyst proteins) in cancer cells. siRNA-mediated knockdown of Sec3 was performed in cancer cells by lipofectamine-based transfection. Post knockdown, cancer cells were stained with CFSE and employed in co-culture with Dil-Ac-LDL stained HUVECs. After 24 h the amount of CFSE transfer was checked by FACS analysis and compared with the control (without Sec3 knockdown) condition. (**d**) Representative image of co-culture showing a reduced amount of TNT formation in the case of Sec3 knockdown of cancer cells. Cancer cells were stained with CellTrace red and co-cultured with unstained HUVECs. After 24 h, the co-culture was fixed, stained with phalloidin green and DAPI, and images were captured with an inverted fluorescence microscope. (**e**) Graph representing a quantification of the number of heterotypic (cancer cell–endothelial cell) and homotypic (cancer cell–cancer cell) nanoscale connections in the control and the Sec3 knockdown condition. The number of homotypic and heterotypic TNTs was counted from at least eight images in each control and knockdown group. The number of cancer cell–endothelial cell (heterotypic) and cancer cell–cancer cell (homotypic) TNT connections in the knockdown group was significantly reduced in comparison to the control group. Data are represented as ±SEM, and an unpaired two-tailed *t*-test was performed with Welch’s correction * *p* = 0.0215 (for cancer–endothelial) and **** *p* < 0.0001 (for cancer–cancer).

The presence of actin filaments in the nanoscale bridges prompted us to investigate the mechanism of cellular cytoskeletal remodeling (actin polymerization) and its involvement in nanobridge formation. The Exocyst–Rho GTPase complex is implicated in actin remodeling and vesicular transport [38,39,40]. We wanted to explore the combined action of the Rho GTPases with selective proteins of the exocyst complex that drive the TNT formation (Figure 2a) [25]. Previous studies have implied the colocalization of Rho GTPase Cdc42 and Rac1 GTPase with the exocyst protein, as well as the TNTs [25]. RalA, a component of the Ral family of GTPase has colocalization with Sec5 and the exocyst protein Sec5 is found throughout TNTs [41,42]. Studies have suggested that RalA also plays an important role in the regulation of exocyst assembly [41].

Further validation of our hypothesis was carried out by performing knockdown of one of the exocyst proteins (Sec3 or EXOC1). siRNA-mediated knockdown was performed in human cancer cells (MDA-MB-231 cells) and validated by immunoblotting (Supplementary Figure S5a). Figure 2b presents the experimental setup, in which the cancer cells (Sec3 knockdown) were stained with CellTrace green and added to Dil-Ac-LDL-stained preformed endothelium. After 24 h, the co-culture of cells was analyzed by FACS. A significantly reduced transfer of cytoplasmic contents was observed in the case of knockdown cells (Figure 2c and Supplementary Figure S5b). The fluorescence microscopy images revealed a lesser amount of nanotube formation between the cancer cells and the endothelial cells in case of Sec3 knockdown (Figure 2d and Supplementary Figure S5c). To gain a quantitative insight into the TNT reduction with Sec3 knockdown, the homotypic and heterotypic TNTs were calculated (Supplementary Figure S5c). Significant reductions in the numbers of nanotubes formed between cancer cell–endothelial cell and cancer cell–cancer cell were observed when Sec3 knockdown was performed (Figure 2e). This further strengthens our theory regarding the involvement of the exocyst-GTPase complex in TNT formation, and offers the opportunity to actively target the same using pharmacological inhibitors.

### 2.3. Action of Pharmacological Inhibition in Intercellular Transfer

**Figure 3:**
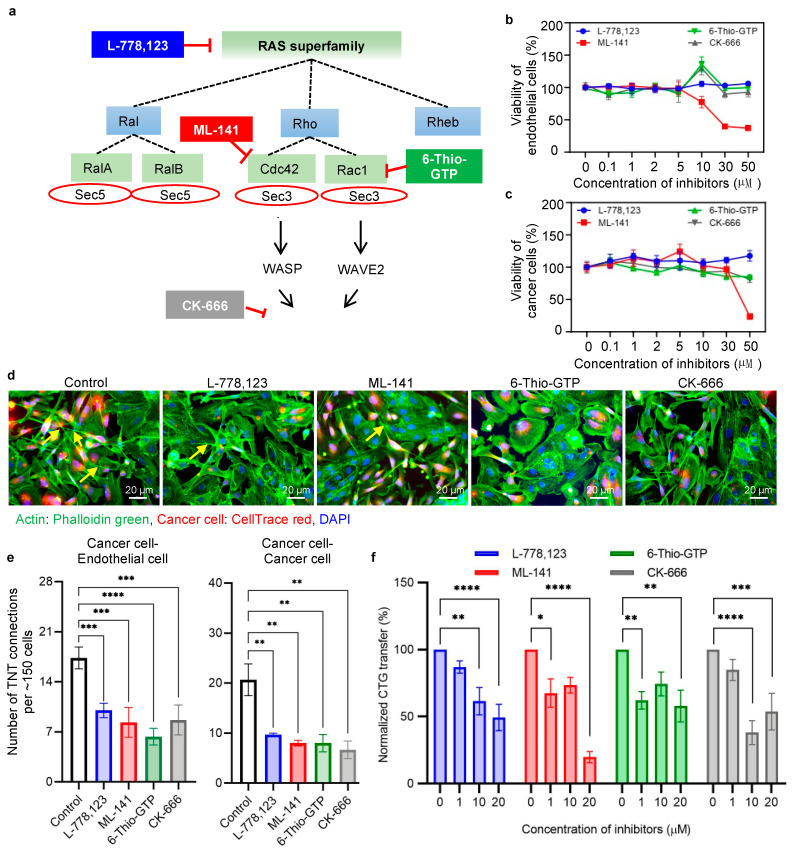
Effect of RAS GTPase inhibitors on TNT formation and CFSE transfer. (**a**) Schematic representation of the signaling cascade of the small Ras family GTPases and exocyst proteins involved in actin polymerization. Specific inhibitors were selected to downregulate proteins in different stages of the signaling pathway. A geranylgeranyltransferase-1 inhibitor, L-778,123, potentially inhibits K-Ras prenylation, ML-141 inhibits activation of Cdc42 GTPase, 6-Thio-GTP inhibits Rac1 GTPase activation, and CK-666 blocks the dimerization of Arp2 and Arp3, which is involved in the formation of new actin filaments. (**b**) Cell viability of endothelial cells in the presence of inhibitors. Cells were treated with different concentrations of inhibitors 0.1–50 µM for 24 h, and the cell viability was measured by MTT assay. The only significant reduction in cell viability was observed at 30 µM and 50 µM concentrations of ML-141 in case of endothelial cells. (**c**) Similarly, cancer cell viability was checked in the presence of inhibitors. Only a concentration of 50 µM of ML-141 was found to be toxic in cancer cells. (**d**) Representative fluorescence microscopy images show the effect of inhibitors in nanoscale communication between cancer cells and endothelial cells. Cancer cells (MDA-MB-231) were loaded with CellTrace red and co-cultured with preformed endothelium in 3D tumor matrix, in the presence and absence of inhibitors. Each inhibitor (L-778,123, ML-141 and 6-Thio-GTP) was used separately at a concentration of 10 μM (except 40 μM for CK-666). The co-culture was stained with phalloidin green to visualize the nanotubes. A visible difference in the number of nanoscale communications was observed in the presence of inhibitors. (**e**) Graph representing the quantification of the number of heterotypic (cancer cell–endothelial cell) and homotypic (cancer cell–cancer cell) nanoscale connections in the control compared with inhibitor-treated conditions. Five images (per experiment) were taken per condition (vehicle control, L-778,123, ML-141 and 6-Thio-GTP, each 10 µM concentration and CK-666 40 µM concentration), with ~150 cells in total being examined, and the number of cancer cell–cancer cell and cancer cell–endothelial cell TNTs formed was counted. The graph shows a significant reduction in the number of TNTs in the presence of inhibitors. The entire experiment was repeated three times (*n* = 3), and the data are presented as mean ±SEM. One-way ANOVA with Dunnett’s multiple comparisons test was performed for statistical analysis, and the following significance values were considered: * *p* < 0.05, ** *p* < 0.01, *** *p* < 0.001. (**f**) Representative bar plot of the dose-dependent reduction of cytoplasmic component transfer from cancer cells to endothelial cells in the presence of inhibitors. Cancer cells (MDA-MB-231) and endothelial cells (HUVECs) were loaded with CellTrace green (CFSE) and Dil-Ac-LDL, respectively, and employed in the co-culture setup in a 3D tumor matrix. Then, inhibitors were added to the co-culture in different concentrations. The transfer of CFSE-labeled cytoplasmic components from cancer cells to endothelial cells was evaluated using a flow cytometer after 24 h of co-culture. The bar plot represents the number of transfers of CFSE to endothelial cells from cancer cells. The experiment was repeated three times (*n* = 3) and the data were normalized with respect to 0 μM concentration and are presented as mean ±SEM. Two-way ANOVA with Dunnett’s multiple comparisons test was performed for statistical analysis, and the following significance values were considered: * *p* < 0.05, ** *p* < 0.01, *** *p* < 0.001, **** *p* < 0.0001.

To evaluate the importance of Rho GTPases on nanotube formation and cellular communication, pharmacological inhibition was performed on several small GTPases involved in the actin polymerization pathway. A simplified schematic representation of the actin polymerization and the involvement of the GTPases and exocyst complex is presented in Figure 2a and Figure 3a. We used a geranylgeranyl transferase inhibitor, L-778,123, which inhibits the geranylation of the K-Ras superfamily, of which small Rho GTPases are subfamilies. Since all these proteins play an important role in actin cytoskeletal remodeling, a permissible concentration was selected by performing titration studies using a cell viability assay. The cell viability of all the above-mentioned inhibitors was checked in both cancer cells and endothelial cells by means of MTT assay. ML-141 (Cdc42 inhibitor) was toxic at concentrations ≥ 30 µM in cancer cells and at 50 µM in endothelial cells. Other inhibitors (L-778,123; 6-Thio-GTP, and CK-666) showed no toxicity until a concentration of 50 μM (Figure 3b,c). Next, we checked the effect of inhibitors on specific proteins and their activation using Western blot analysis (Supplementary Figure S6a–c). For the geranylgeranyl transferase inhibitor, L-778,123, we checked the expression of K-Ras using a prenylation-specific K-Ras antibody. At 10 µM and 20 µM concentrations of L-778,123, the expression was considerably reduced, along with the reduction in the downstream protein WAVE2 (Supplementary Figure S6a). Additionally, in the case of ML-141, a major reduction in downstream protein WASP was observed (Supplementary Figure S6b). A greater degree of reduction in WAVE2, the downstream protein of Rac1, was observed in the case of the 6-Thio-GTP inhibitor (Supplementary Figure S6c). The inhibitors were applied in the co-culture of cancer cells and endothelial cells, and a significant reduction of TNT formation was observed (Figure 3d). Endothelial cells were grown in the 3D tumor matrix to form the endothelial network. The co-culture was set up between CellTrace red-stained cancer cells and unstained endothelial cells in the presence of GTPase inhibitors (10 µM for L-778,123, ML-141 and 6-Thio-GTP, and 40 µM for CK-666). After 24 h of co-culture, the cells were fixed, stained with phalloidin green and DAPI, and imaged using a fluorescence microscope. Reduced number of TNT formations was visible in the inhibitor-treated co-culture (Figure 3d). Both the cancer cell–endothelial cell heterotypic connections and the cancer cell–cancer cell homotypic connections were greatly reduced in the presence of inhibitors (Figure 3e and Supplementary Figure S7a–e). Quantification of TNTs was done in accordance with the TNT counting method, as explained in Supplementary Figure S8a–c.

Moreover, the transfers of cytoplasmic components were also reduced in the presence of the GTPase inhibitors. Cancer cells loaded with CFSE were added to a preformed endothelial network (Dil-Ac-LDL stained) in a 3D tumor matrix, and the transfer of CFSE-labeled cellular components was measured using flow cytometric analysis (Supplementary Figure S9). The dose-dependent reduction of intercellular transfer of cytoplasmic contents from cancer cells to the endothelial cells was observed in the presence of the GTPase inhibitors (Figure 3f). At concentrations of 10 µM and 20 µM, all the inhibitors showed a significant decrease in the percentage of endothelial cells having CFSE from cancer cells. These data demonstrate a correlation between the inhibitors and intercellular communication.

To strengthen our hypothesis, the action of inhibitors in other highly metastatic cancer cell lines—MDA-MB-468 and PC-3—was also observed. Endothelial cells were grown in the 3D tumor matrix to form the endothelial network. The co-culture was set up between CFSE-stained cancer cells (MDA-MB-468 and PC-3 cells) and tdTomato-labeled endothelial cells in the presence of GTPase inhibitors (10 µM for L-778,123, ML-141, and 6-Thio-GTP; and 40 µM for CK-666). After 24 h of co-culture, the cells were fixed, stained with rhodamine phalloidin and DAPI, and then imaged using a fluorescence microscope. A similar visible reduction in nanotubular structures was observed in both cell lines (MDA-MB-468 and PC-3 cancer cells) (Supplementary Figure S10, images for red and green are pseudo colored to maintain the continuity of all the images throughout the manuscript). A similar co-culture setup was implemented to evaluate the action of inhibitors in the transfer of cytoplasmic components from cancer cells to endothelial cells. The reduction in CFSE transfer from the MDA-MB-468 and PC-3 cells to the endothelial cells followed the same pattern as that observed for MDA-MB-231 cells (Supplementary Figure S11).

### 2.4. Inhibiting Actin Polymerization Pathway Leads to a Reversal of Phenotypic Transition

**Figure 4:**
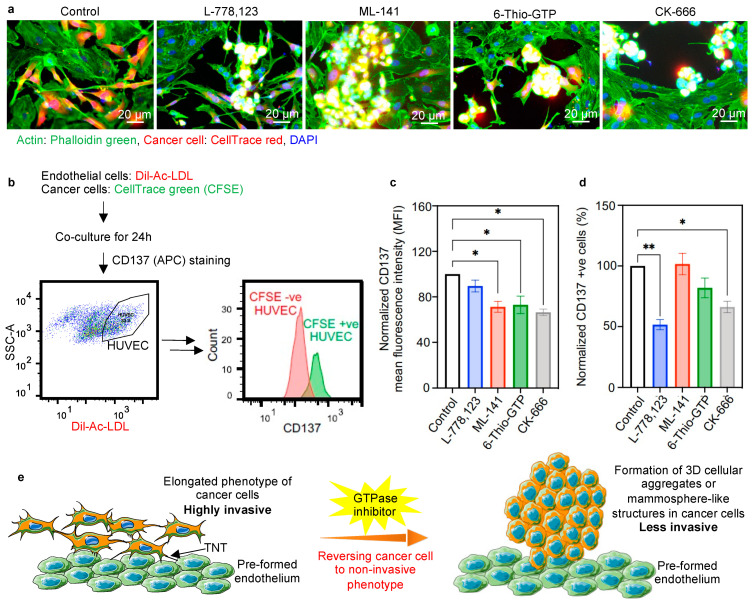
Reversal of phenotypic transition in the presence of inhibitors. (**a**) Representative images of co-culture of cancer cells and endothelial cells in the 3D tumor matrix, showing the reversal of the phenotypic transition of cancer cells from a highly invasive elongated phenotype to 3D cellular aggregates or mammosphere-like structures with the effect of inhibitors. Cancer cells (MDA-MB-231) were stained with CellTrace red and co-cultured with unstained endothelial cells. Co-cultures were set up in the absence and presence of inhibitors (10 μM each, 40 μM for CK-666). After 24 h of co-culture, the cells were stained with phalloidin green; nuclei were counterstained with DAPI and imaged under a fluorescence microscope. The mammosphere formation (yellow signal) was observed in the cancer cells in the presence of inhibitors. (**b**) Experimental design for checking the relative expression of tumor endothelial markers in HUVECs after being involved in nanoscale communication with cancer cells. HUVECs were stained with Dil-Ac-LDL and co-cultured with CFSE stained cancer cells (MDA-MB-231). The histogram showing CFSE +ve HUVECs, i.e., the cells that received cytoplasmic components from cancer cells, exhibited higher CD137 expression than CFSE-ve HUVECs. (**c**) Representative bar plot showing CD137 mean fluorescence intensity (MFI) of CFSE +ve HUVECs in the presence of GTPase inhibitors. A significant reduction of CD137 MFI was observed that signifies the reduced transfer of information from cancer cells to endothelial cells. (**d**) Bar plot showing the comparative population of CFSE +ve endothelial cell (Dil-Ac-LDL +ve) with high CD137 expression. A significant reduction in the number of cells expressing high CD137 was observed upon inhibitor treatment. This signifies the reduced transfer of information from cancer cells to endothelial cells. (**e**) Schematic representation of the effect of inhibitors on the phenotype transition of cancer cells. Metastatic cancer cells adopt an elongated phenotype in the presence of the endothelial layer. The elongated phenotype of cancer cells is able to participate in active communication with endothelial cells, and can easily invade the endothelial layer, resulting in enhanced metastasis. In the presence of GTPase inhibitors, cancer cells return to their original 3D cellular aggregates or mammosphere-like phenotypes, which are less invasive, and which correlate with cancer cells with a reduced chance of invasiveness. * *p* < 0.05, ** *p* < 0.01.

GTPase inhibitors were also capable of reversing the more invasive phenotype of cancer cells into the less invasive mammospheres. As explained in Figure 1a-d, the cancer cells adopted an elongated and more invasive phenotype when co-cultured in the presence of endothelium. In contrast to that, we observed the reversal of the cancer cell phenotype into less invasive 3D cellular aggregates or mammosphere-like structures when co-cultured with endothelium in the presence of inhibitors (Figure 4a). Similar cellular aggregates or mammosphere-like structures were also observed for the other metastatic cancer cell lines (MDA-MB-468 and PC-3 cells) upon the action of inhibitors (Supplementary Figure S10).

Since our study revealed that the inhibitors were able to change the highly invasive phenotype of cancer cells into a less invasive one in the presence of endothelium, we wanted to look more closely at the endothelial phenotype when co-cultured with cancer cells in the presence of inhibitors. In our previous report, we demonstrated the increased expression of tumor-associated endothelial markers [7], like CD137, in the tumor blood vessel walls [43].

To check the expression of CD137 in endothelial cells in the presence of inhibitors, we first screened CD137 expression in endothelial cells, which were involved in the TNT communication and accepted CFSE from cancer cells (Figure 4b). CFSE-labeled cancer cells were co-cultured with Dil-Ac-LDL-positive endothelial cells, and after 24 h, the cells were stained with CD137 and analyzed by means of flow cytometry. The CFSE +ve subpopulation of endothelial cells (Dil-Ac-LDL) represents the fraction of endothelial cells involved in TNT-mediated communication, and which accepts CFSE from cancer cells. Figure 4b represents a population of endothelial cells separated on the basis of the presence of CFSE after the occurrence of physical communication with the CFSE-loaded cancer cells (Supplementary Figure S12). The CD137 expression in the CFSE recipient cells (Dil-Ac-LDL +ve, CFSE +ve) was significantly reduced in the presence of inhibitors. The mean fluorescence intensity (MFI) of CD137 exhibited a significant reduction in the presence of ML-141, 6-Thio-GTP, and CK-666 (Figure 4c). Additionally, the number of endothelial cells expressing CD137^high^ was significantly reduced in the presence of L-778,123 and CK-666 (Figure 4d). These data indicate the reduction of the tumor-associated endothelial phenotype in the presence of GTPase inhibitors.

## 3. Discussion

The cross talk between cancer cells and the endothelium is one of the major events in the case of cancer metastasis. Previously, we have demonstrated that cancer cells establish nanotubular communication with the endothelial cells and transfer microRNAs to convert the endothelium into a pathological one [7]. We also demonstrated the phenotypic plasticity of the cancer cells during communication with endothelial cells. Metastatic human cancer cells transform their phenotype from cellular aggregates or mammosphere-like structures to an elongated phenotype when cultured with endothelial cells in 3D tumor matrix [9]. Here, we demonstrate that disrupting the nanotubes can revert this invasive phenotype to a non-invasive phenotype.

The mechanistic study of TNT formation has not been clearly discussed in the literature with respect to cancer metastasis. We found that the TNTs between the cancer cells and the endothelial cells were primarily composed of actin cytoskeletal elements. This opens the possibility of exploring the participation of the actin polymerization pathway in TNT formation. The involvement of the GTPase–exocyst complex in the dynamics of membrane–actin is a potential target for investigating the mechanism of TNT formation. To strengthen this hypothesis, we first performed siRNA-mediated knockdown of Sec3 (EXOC1) in the cancer cells, which showed a significant reduction in nanotubular cancer cell–endothelial cell and cancer cell–cancer cell connections. The transfer of CFSE from cancer to endothelial cells was also reduced in the case of Sec3 knockdown cancer cells. These observations signify the involvement of the GTPase–exocyst complex in the TNT-mediated communication between cancer and endothelial cells. Additionally, this opens up the possibility of introducing pharmacological inhibitors for GTPase-exocyst GTPase–exocyst complex in order to reduce the physical communication between highly metastatic cancer cells and the endothelium.

The Rho GTPases are a prime target for pharmacological inhibitors, as exocyst complex inhibitors have not been well studied in the literature. To inhibit the exocyst–GTPase complex, several pharmacological inhibitors for GTPases were selected on the basis of their inhibitory effect in different stages of the actin polymerization pathway. Indeed, the GTPases take part in fulfilling the energy requirement during actin remodeling, which is a key step in the rapid cell proliferation of cancer cells.

Most importantly, Rho-family GTPases act as GTP-dependent molecular switches to regulate the restructuring of the actin cytoskeleton [44]. Among the small GTPases of the Rho family, Cdc42 and Rac1 play a major role in actin assembly, directing the formation of filopodia, lamellipodia and stress fibers [45]. We tested the effect of pharmacological inhibitors like ML-141 and 6-Thio-GTP, targeting specifically the Rho GTPases, Cdc42 and Rac1, respectively. We also checked the effect of actin nucleation factor Arp2/3, which is activated by GTPases downstream of Cdc42 or Rac1 called WASP or WAVE2, respectively [46]. We used the CK-666 inhibitor for the Arp2/3 actin nucleation factor. We took a geranylgeranyl transferase inhibitor, L-778,123, which inhibits the geranylation of the K-Ras superfamily, under which the subfamilies are small Rho GTPases. GTPases require carboxy-terminal geranylation and membrane association for biological activity, and therefore a pharmacological inhibition of geranylation can disrupt their activity [40,47]. Since all these proteins play an important role in actin cytoskeletal remodeling, a permissible concentration was used by checking the cell viability for each inhibitor for each type of cells. Use of the inhibitors in the co-culture significantly reduced the homotypic and heterotypic TNT communications. A dose-dependent reduction of CFSE transfer was also observed in the case of all inhibitors. Hence, the inhibitors are capable of blocking direct physical communication between the cancer cells and the endothelial cells without loss of cell viability.

The 3D cellular aggregates or mammosphere-like structures of cancer cells transformed to an elongated spindle-like structure when interacting with the endothelium in the 3D tumor matrix. The direct physical communication between cancer cells and endothelial cells imposes the phenotypic change, and thereby facilitates metastasis. Indeed, in the presence of Rho GTPase inhibitors, the cancer cells reverted to less invasive cellular aggregates or mammosphere-like structures, implicating TNT-mediated communication between the cancer cells with the endothelial cells in the phenotypic transition (Figure 4e). Moreover, knockdown of the Sec3 exocyst protein prompted a similar reversion to the mammosphere-like structure. Hence, the results signify the importance of the exocyst–Rho GTPase complex in maintaining the physical nanoscale communication for regulating the invasiveness of tumor cells. These results indicate that the tumor cells and endothelial cells interacting through those TNT communications create an environment helpful in obtaining an invasive phenotype in cancer cells.

The expression of tumor-associated endothelial marker CD137, which is highly expressed in the pathological tumor blood vessel walls [43], was highly increased in the CFSE recipient endothelial cells (Dil-Ac-LDL +ve, CFSE +ve). In the presence of inhibitors, the CD137 expression in the CFSE recipient endothelial cells was significantly reduced. Hence, the phenotypic transition of endothelial cells to a pathological one resulting from the TNT-mediated communication between cancer cells and endothelial cells is also reduced in the presence of the pharmacological inhibitor. Our results suggest that the GTPase inhibitors can inhibit the TNT-mediated communications and restrict the phenotypic transitions favorable for cancer metastasis. The use of these GTPaes inhibitors could be a potential target for next-generation treatment of cancer metastasis.

## 4. Materials and Methods

### 4.1. Reagents

Dulbecco’s modified Eagle’s medium (DMEM), Dulbecco’s phosphate-buffered saline (DPBS), fetal bovine serum (FBS), trypsin/EDTA, trypan blue stain (0.4%) and ethylenediamine tetra-acetic acid (EDTA 0.5 M, pH 8) were obtained from Gibco, Thermo Fisher, Waltham, MA, USA.

### 4.2. Cell Culture

Human umbilical vein endothelial cells (HUVECs) (Lonza, Morristown, NJ, USA) were cultured on a 0.1% gelatin-coated tissue culture flask in EBM-2 medium (Lonza) supplemented with a bullet kit (Lonza). MDA-MB-231 (ATCC, Manassas, VA, USA) and MDA-MB-468 (ATCC) highly metastatic human breast cancer cells, MCF-7 (ATCC) poorly metastatic human breast cancer cells and PC-3 (ATCC) human pancreatic cancer cells were cultured in DMEM (Dulbecco’s modified Eagle’s medium) supplemented with 10% fetal bovine serum (FBS) and 1% *v/v* Penicillin-Streptomycin-Glutamine (PSG). All cells were mycoplasma free.

### 4.3. Co-Culture

Endothelial cells were incubated with Dil-Ac-LDL (1:100) (Cell Applications, San Diego, CA, USA) in complete media overnight, and plated in EBM-2 complete media for 5–6 h incubation in pre-coated Matrigel suspension wells (1:2 dilution with PBS). The endothelial cells used in co-culture with MDA-MB-468 and PC-3 cancer cells were transfected with LV-EF1α-tdTOMATO-IRES-Puro, pre-made lentivirus, which expresses tdTomato (SignaGen Laboratories, Frederick, MD, USA). We followed the manufacturer’s protocol to perform the transfection and induce tdTomato expression. Cancer cells were incubated with 1 μM of CellTrace green (CFSE, Life Technologies, Waltham, MA, USA) in DMEM basal media for 1 h. The cells were added to the preformed vessels in their respective complete media in a 1:1 ratio, which was the same as the ratio of cancer cells: endothelial cells and incubated for 24 h before analysis.

### 4.4. Matrix Preparation

Corning^®^ Matrigel^®^ Matrix (Tewksbury, MA, USA) was allowed to thaw at 4 °C and diluted with DPBS in 1:2 dilution, 125 µL of diluted matrix per cm^2^ was added, while preventing the formation of bubbles. The matrix was allowed to solidify at 37 °C in an incubator for more than approximately 1 h, before the cells were seeded on the matrix.

### 4.5. Drug Treatment

For the GTPase inhibition, cancer cells were pre-incubated for 6 h with 10 µM and 20 µM of each geranylgeranyltransferase 1 inhibitor (L-778,123, Bio-vision, Milpitas, CA, USA), Cdc42/Rac1 GTPase Inhibitor (ML141 and 6-Thio-GTP; Sigma, St. Louis, MO, USA), and 10 µM and 40 µM of Arp2/3 complex inhibitor (CK-666, Sigma) in basal media before adding them to the co-culture setup. Following the addition of cancer cells after endothelium formation, inhibitors were added in complete media. Both the fluorescence signals of the labeled cells and their transfer among the cells were measured, and the untreated co-culture and different drug treatment groups were compared on the basis of flow cytometric analysis and fluorescence microscopy imaging.

### 4.6. MTT Assay

To determine the viability of breast cancer cells and endothelial cells in vitro, 10^4^ cells were treated in the presence of various concentrations of GTPase inhibitors, i.e., geranylgeranyltransferase 1 inhibitor (L-778,123), Cdc42/Rac1 GTPase inhibitor (ML-141 and 6-Thio-GTP), and Arp2/3 complex inhibitor (CK-666), and the maximum concentration of 0.2% DMSO (dimethyl sulfoxide) used with the drugs as a vehicle for 24 h. After incubation with drugs, cells were incubated for 4 h with 0.5 mg/mL final concentration of MTT (3-(4,5-dimethylthiazol-2-yl)-2–5-diphenyltetrazolium bromide) reagent in basal media. Formazan crystals were dissolved using an organic solvent DMSO, prior to recording absorbance using a BioTek Epoch Microplate Spectrophotometer (Winooski, VT, USA) at 562 nm. Three replicates were performed per condition, and the experiment was repeated thrice.

### 4.7. siRNA Transfection

Cancer cells were transfected with EXOC1 siRNA (MISSION siRNA, Sigma-Aldrich, St. Louis, MO, USA) and GAPDH control siRNA (Sigma-Aldrich) using Lipofectamine RNAiMAX (Invitrogen, Waltham, MA, USA) according to the manufacturer’s protocol. Briefly, cells (at 90% confluency) were cultured in Opti-MEM I (Invitrogen, Waltham, MA, USA). A siRNA–lipid complex, comprising 2.5 µL (0.1 pM) and 4 µL (0.2 pM) of EXOC1 siRNA in 150 µL of Opti-MEM was mixed with 9 µL of Lipofectamine RNAiMAX in 150 µL of Opti-MEM, was prepared. After incubation for 15 min at room temperature, the cells were incubated for 12 h with siRNA–lipid complex, and then the cells were left in complete media only for the next 36 h. Transfection was validated by Western blot analysis.

### 4.8. Flow Cytometry

Cells were recovered from the 3D tumor matrix by treating the cells with cell recovery solution (Corning), and cell cell suspensions were prepared in PBS with 2% FBS, maintaining a density of 1–5 million cells/mL. Cells were fixed, permeabilized and stained with CD137 (APC) antibody depending on the manufacturers protocol, according to the experimental design. Samples were examined by Accuri C6 Flow Cytometer (San Jose, CA, USA) or BD LSR Fortesa Flow Cytometer (San Jose, CA, USA). Data were processed using FlowJo 10.7.1 software (BD, Ashland, OR, USA).

### 4.9. Fluorescence Microscopy

Cells after co-culture were washed with 1X PBS and were fixed with 4% paraformaldehyde (PFA) at room temperature for 20 min, with subsequent washing post-fixation. Permeabilization was achieved by incubation with 0.5% Triton X-100 at 4 °C for 10 min. After permeabilization, cells were washed with 1X PBS-T (1X PBS + 0.05% Tween 20) followed by blocking with 10% BSA solution or 10% goat serum (dilution with PBS-T) at room temperature for 1 h. F-actin was stained with phalloidin (Alexa Fluor 488 conjugated or Rhodamine Phalloidin) (1:1000) (Life Technologies, Waltham, MA, USA) and incubated at room temperature for 1 h. After incubation, cells were incubated with Hoechst 33,324 to stain the nucleus, and images were then taken using the fluorescence microscope.

### 4.10. TNT Counting

TNT formation was quantified after 24 h of co-culture of MDA-MB-231 cancer cells and preformed endothelium with HUVECs. About 0.2 million cells of each cell type were seeded per well of a 24-well plate. Cells were fixed and stained with Phalloidin to stain the actin filaments. In this study, a TNT was defined as a thin membranous structure, partially non-adherent to the substratum, with a diameter ≤200 nm, and at least ≥5 µm in length. Five images were taken per coverslip of each condition, TNT was counted to consider ~150 cells in total, and the experiment was repeated thrice (*n* = 3). A clear example of TNT counting is demonstrated in the Supplementary Materials, in which all TNTs are shown partially above the substratum.

### 4.11. Western Blotting

Cells were seeded at a density of 2–3 × 10^6^ cells in a T-25 flask. The treatments of drug/inhibitor were performed according to the experimental design, with equal amounts of DMSO (<0.1%) in each sample. Cells were lysed with RIPA lysis buffer supplemented with protease and phosphatase inhibitor for 30 min on ice with mild vortexing every 10 min followed by centrifugation at 4 °C. The amount of protein was measured by BCA assay (Thermo Fisher Scientific) (according to the supplier’s protocols) and an equal amount of protein lysates was electrophoresed on a 10–15% polyacrylamide gel at 80–140 volts for 1 h. The proteins were transferred to nitrocellulose or polyvinylidene difluoride (PVDF) membrane (0.2 µm pore size) through wet blotting for 3 h at 250 mAmp (Biorad, Billerica, MA, USA). Blocking was performed using 5% BSA in 1x TBS-T (1X TBS 0.1% TWEEN-20) for 1 h, and then the membrane was incubated with primary antibodies at 4 °C overnight. After that, a washing process was performed three times with 1x TBS-T, and the samples were incubated with secondary antibody HRP-goat-anti-rabbit/mouse (1:3000 for GAPDH and 1:2000 for other proteins) (BioRad) for 1 h at RT. Then, membranes were washed thrice with 1X TBS-T and developed using a femto chemiluminescent substrate (Thermo Scientific) and developed using G Box Bio Imaging system (Syngene, Frederick, MD, USA), and the image analysis was carried out using ImageJ 1.52a software (NIH, Bethesda, MD, USA). Antibodies for Western blotting: WAVE2 (CST, 1:1000), WASP (CST, 1:1000), Cdc42 (SCBT, 1:200), Rac1 (SCBT, 1:100), anti c-k-Ras (SCBT, 1:100) and GAPDH (SCBT, 1:2000).

## 5. Conclusions

This study provides new insights into the treatment of tumor metastasis by reversing the invasiveness of the tumor cells. The observations of TNT reduction by pharmacological inhibition of Rho GTPases and knockdown of the exocyst complex suggests potential involvement of these proteins in TNT formation. The use of pharmacological inhibitors specifically reduced the transfer of cytoplasmic elements among the cancer cells and the endothelial cells and reversed the phenotypic transition occurred due to the communication of both cancer cells and endothelial cells. Inhibition of the physical communication between cancer cell and endothelial cell can potentially reduce the chance of tumor invasion and metastasis. A detailed study of those inhibitors on cancer metastasis is in progress.

## Data Availability

All data are available in the manuscript and supplementary materials.

## Supplementary Materials

Supplementary materials can be found at https://www.mdpi.com/article/10.3390/ijms22116161/s1.
