# Supplementary material for: Inhibition of Tunneling Nanotubes between Cancer Cell and the Endothelium Alters the Metastatic Phenotype
Source: Int J Mol Sci. 2021 Jun 7;22(11):6161. doi: 10.3390/ijms22116161 (PMC8200952; doi:10.3390/ijms22116161)
Supplement: Supplementary file 1 [file ijms-22-06161-s001.zip › ijms-1191025-SI.pdf]

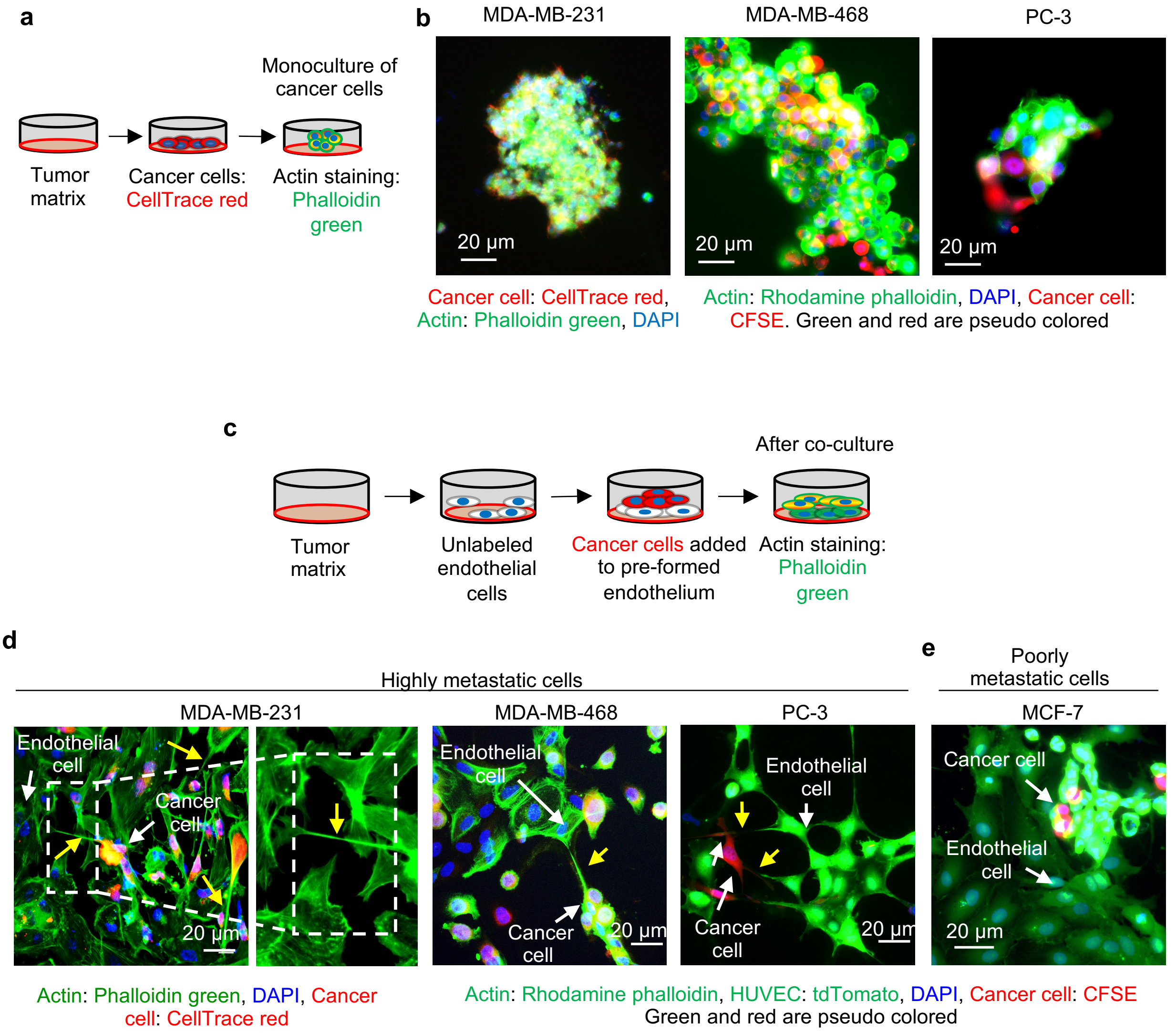

Supplementary Figure S1: (**a, b**) Schematic of the experimental design and representative images of the formation of three dimensional mammosphere-like structures or cellular aggregates for monoculture of each cancer cell lines (MDA-MB-231, MDA-MB-468 and PC-3 cells) in the 3D tumor matrix in the absence of endothelium. (**c, d**) Schematic showing the experimental design and representative images for co-culture of each cancer cell types (highly metastatic cell lines MDA-MB-231, MDA-MB-468 and PC-3 cells) with preformed endothelium of HUVECs for 24 h in the 3D tumor matrix. MDA-MB-231 cancer cells, loaded with CellTrace red were used in the co-culture with endothelial cells. In case of MDA-MB-468, PC-3 and MCF-7 cells, the cancer cells are stained with CFSE, and endothelial cells have tdTomato expression (Red and green are pseudo colored to maintain the continuity of images in the whole manuscript). After 24h of co-culture cells were fixed, actin filaments were stained with phalloidin green (rhodamine phalloidin in case of MDA-MB-468, PC-3 and MCF-7 cancer cells), and nuclei were counterstained with DAPI. In the images, the green signal is from the phalloidin staining (which stain both cancer and endothelial cell), and red signal is from cancer cells. Yellow arrows indicates the tunneling nanotubes (or TNTs) formed between the cancer cell and the endothelial cell. The magnified image with MDA-MB-231 cancer cells representing the presence of actin filaments in the nanoscale structures. (**e**) Representative image for co-culture of HUVECs and poorly metastatic cell line MCF-7. No TNT formation was observed between the MCF-7 cancer cells with the endothelial network. The MCF-7 cells were found to form 3D cellular aggregates or mammosphere-like structures even in the co-culture, probably due to their less invasive property.

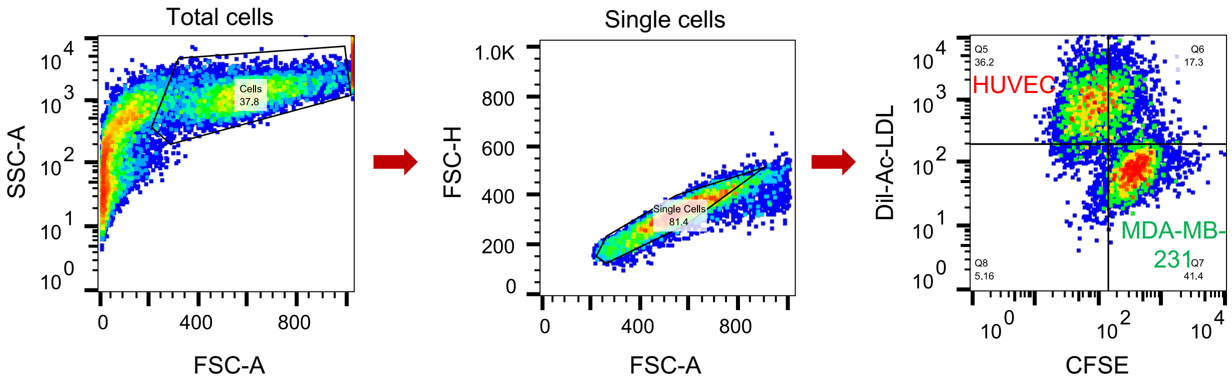

Supplementary Figure S2: The representative gating strategy of flow cytometric analysis to monitor the CFSE transfer from cancer cells to endothelial cells. MDA-MB-231 cancer cells (CFSE) were co-cultured with HUVECs (Dil-Ac-LDL) for 24 h. The third plot signifies the presence of CFSE stained cancer cells in the lower right corner, Dil-Ac-LDL-stained endothelial cells in upper left corner and the endothelial cells that have taken up CFSE are in the upper right corner.

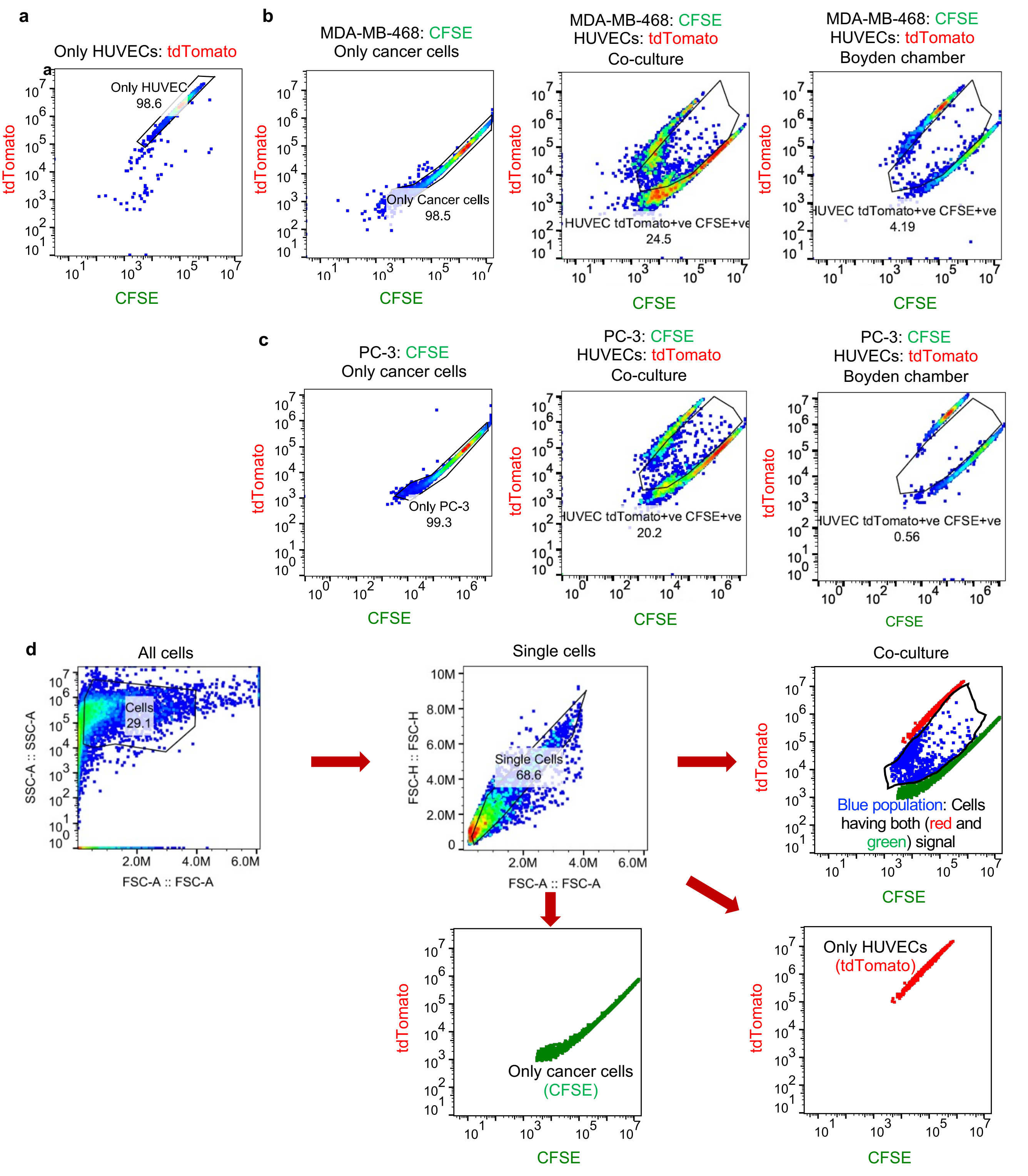

Supplementary Figure S3: The transfer of cellular cytoplasmic components (CFSE) from different cancer cell lines (MDA-MB-468 and PC-3 cells) to endothelial cells. Cancer cells (MDA-MB-468 and PC-3 cells) stained with CFSE were co-cultured with HUVECs (tdTomato) for 24 h in the 3D tumor matrix. The direct co-culture setup was also compared in a co-culture setup in Boyden chamber, where the cancer cells and endothelial cells were added to upper and lower compartment, respectively, and separated by a membrane of 0.4 µm pore size. Hence the direct nanoscale communication between cells was forbidden, whereas the exosomal and paracrine communications are allowed. FACS plot representing the monoculture of each cell type, (**a**) HUVECs (tdTomato) and (**b, c**) MDA-MB-468 and PC-3 cancer cells (both CFSE), in the respective fluorescence channel, co-culture showing the CFSE transfer from MDA-MB-468 and PC-3 cancer cells to endothelial cells by 24.5% and 20.7% and co-culture in Boyden chamber has lesser CFSE transfer to endothelial cells with 4.19% and 0.56% respectively. (**d**) The representative gating strategy of flow cytometric analysis to monitor the CFSE transfer from cancer cells to tdTomato positive endothelial cells, which are co-cultured for 24h in the 3D tumor matrix. The population corresponding to cancer cells (CFSE) and endothelial cells (tdTomato) were color-coded according to the fluorescence signal. The blue population represents the cells having both green and red fluorescence.

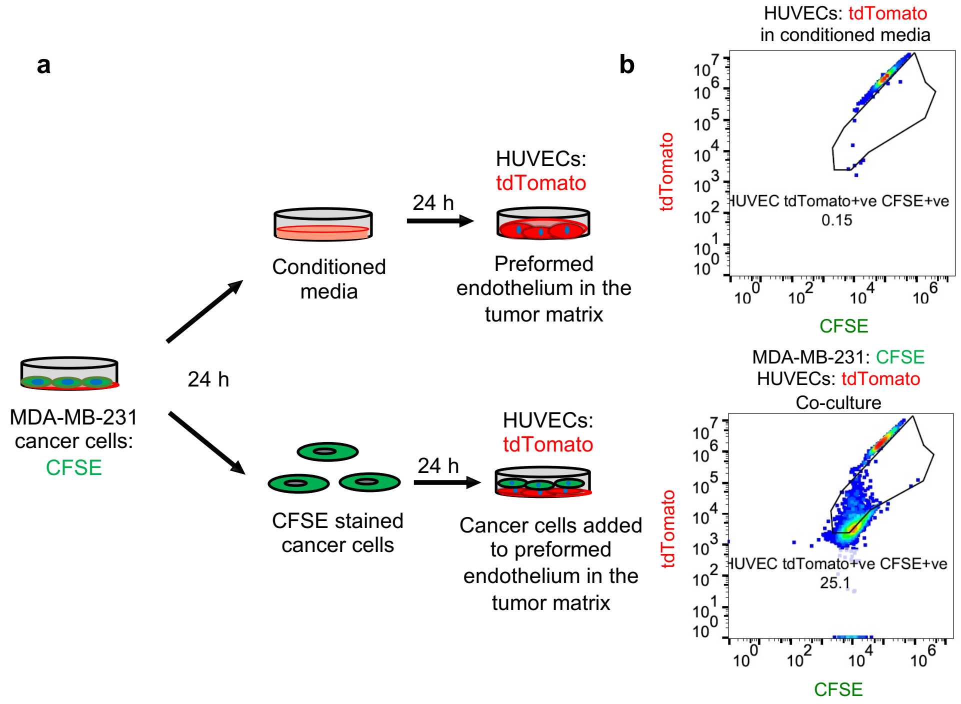

Supplementary Figure S4: Control study to test for CellTrace green (CFSE) dye leakage from cancer cells contributing to labeling of endothelial cells. (**a**) Schematic representation of the conditioned media experiment. MDA-MB-231 cancer cells were stained with CellTrace green or CFSE (1 μM), washed thoroughly as mentioned in the methods section, and cultured in complete media for 24 h. The conditioned media of cancer cells was collected and added to preformed endothelium with tdTomato labeled HUVECs for another 24 h in the 3D tumor matrix. On the other hand, the CFSE stained MDA-MB-231 cancer cells were co-cultured with tdTomato labeled HUVECs in the tumor matrix for 24 h. Exact same amount of media has been used to avoid any dilution effect in the experiment. (**b**) Scatter plot showing absence of CFSE staining in the HUVECs cultured in conditioned media of cancer cells (upper plot). The HUVECs co-cultured with CFSE labeled cancer cells showed transfer of cytoplasmic components to HUVECs (lower plot). The gating strategy was followed as described in Supplementary Fig. 3d.

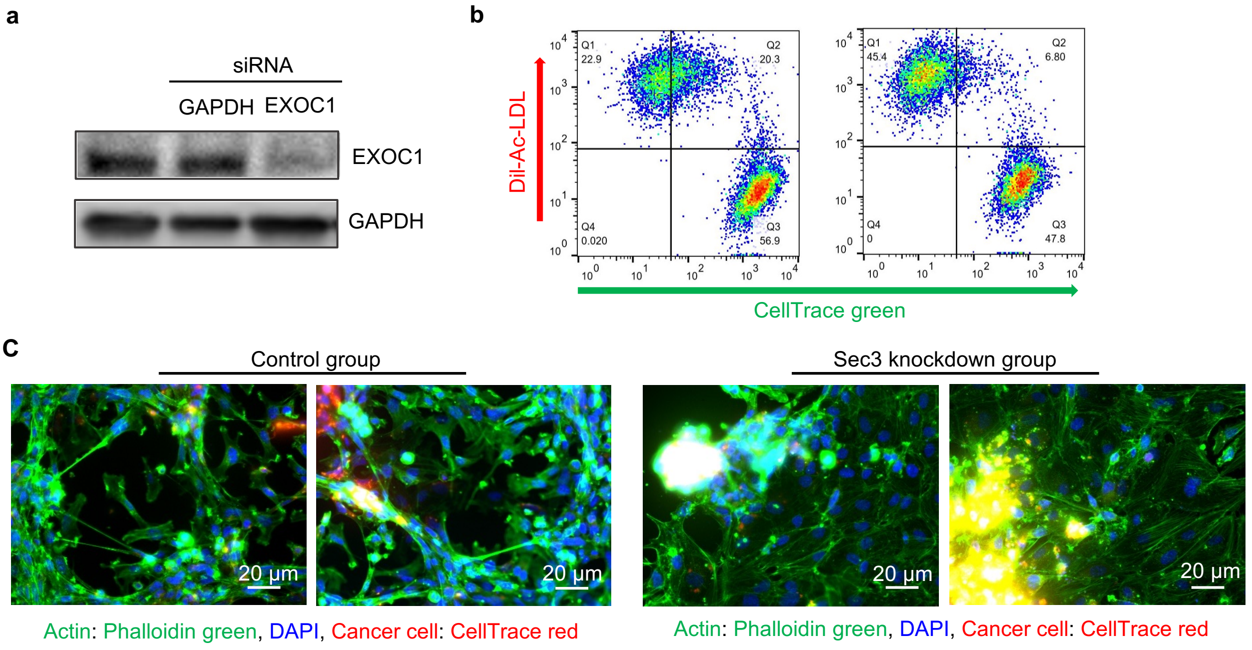

Supplementary Figure S5: (**a**) Immunoblots to check the knockdown of Sec3 (EXOC1) compared with GAPDH expression. Sec3 siRNA and GAPDH siRNA (as a control) were loaded in lipofectamine vehicle and added to the cancer cells. After 12 h, the cancer cells were washed and replaced with fresh media. The amount of EXOC1 and GAPDH were identified by immunoblot analysis after 48 h of culture. (**b**) Representative FACS plots show CFSE transfer in co-culture (vehicle control) of 20.3% which has been reduced in case of using transfected cancer cells with Sec3 siRNA in the co-culture to 6.8%. Cancer cells stained with CellTrace green (CFSE) and endothelial cells with Dil-Ac-LDL co-cultured for 24 h in the 3D tumor matrix. (**c**) Representative image of co-culture showing reduced number of TNT formation in case of Sec3 knockdown. siRNA mediated knockdown was performed in cancer cells (MDA-MB-231), and then, co-cultured with HUVECs. Cancer cells were stained with CellTrace red before placing the co-culture. After 24 h of co-culture, the cells were fixed, stained with phalloidin green and images were captured with inverted fluorescence microscope.

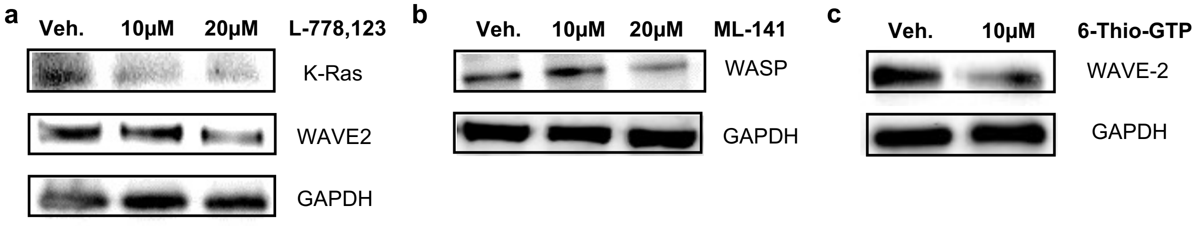

Supplementary Figure S6: Representative immunoblots show dose dependent reduction of corresponding protein expression in presence of various inhibitors. MDA-MB-231 cancer cells were cultured in presence of the specific inhibitors for 24 h, and then, cell lysates were prepared. Immunoblot analysis was performed by using corresponding primary antibody with HRP-conjugated secondary antibody. (**a**) K-Ras prenylation and WAVE2 expression levels were checked for L-778,123 inhibitor. The used K-Ras antibody is specific to recognize the prenylated form of K-Ras. WAVE2 come in the downstream signaling of K-Ras. (**b**) Dose dependent expression of WASP, a downstream signaling protein of Cdc42 in presence of ML-141 inhibitor (0, 10, 20 μM). (**c**) WAVE2 expression for 6-Thio-GTP.

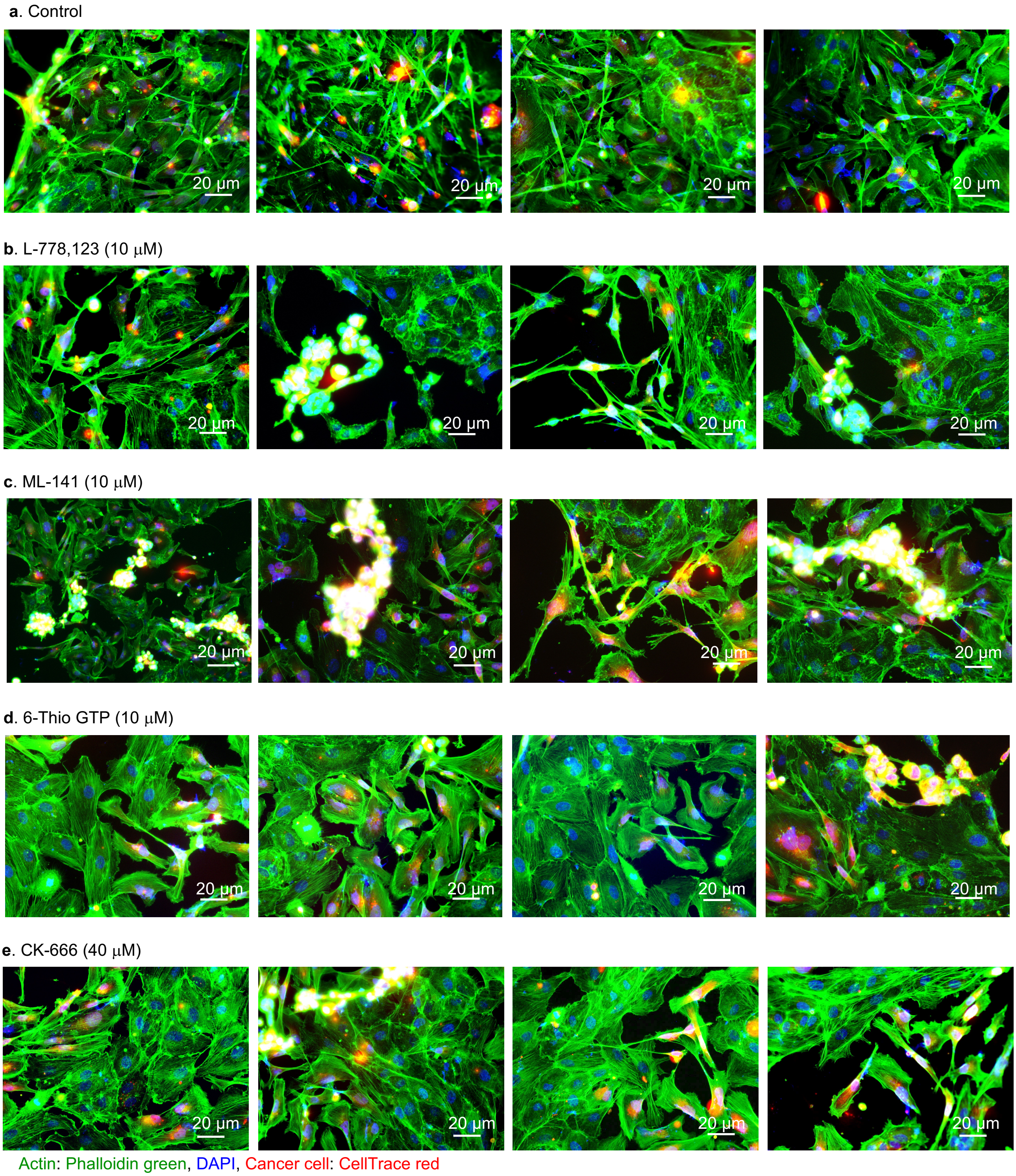

Supplementary Figure S7: (**a-e**) Representative fluorescence microscopy images show the effect of inhibitors in nanoscale communication between the cancer cells and the endothelial cells. Cancer cells (MDA-MB-231 cells) were loaded with CellTrace red and co-cultured with preformed endothelium in tumor matrix, in presence and absence of inhibitors. Each inhibitor (L-778,123, ML-141, 6-Thio-GTP and CK-666) was used separately at a concentration of 10 µM (40 µM for 6-Thio-GTP). The co-culture was stained with phalloidin green to visualize the nanotubes. A visible difference in number of nanoscale communications was observed in presence of inhibitor. This data signifies the importance of the Ras family GTPases in actin polymerization and formation of TNTs. At least five images were recorded for each condition in every experiment. The experiment was repeated in triplicate. Only four representative images in each condition are shown here.

a **b**

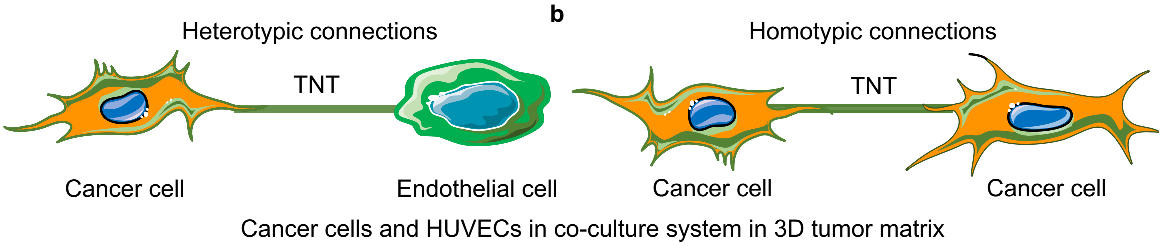

c

TNT counting:

- Partially non-adherent to the substratum
- ≤ 200 nm of diameter and at least ≥ 5 μm length
- TNTs were counted for considering ~150 cells in total and the experiment has been repeated thrice (n=3).

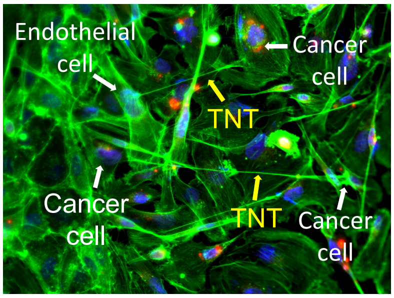

Actin: Phalloidin green, DAPI, Cancer cell: CellTrace red

Supplementary Figure S8: (**a, b**) Schematic representation of the formation of TNT in the co-culture system. TNTs are formed between the cancer cell and the endothelial cell (heterotypic connections) and in between the cancer cells (homotypic connections). (**c**) A representative image showing the TNTs formed between cancer cell and endothelial cells (yellow arrows) and between the cancer cells (purple arrows) with a detailed explanation of the method of TNT counting from images. Cancer cells loaded with CellTrace red were used in the co-culture with endothelial cells. After 24h of co-culture, cells were fixed, actin filaments were stained with phalloidin green, and nuclei were counterstained with DAPI.

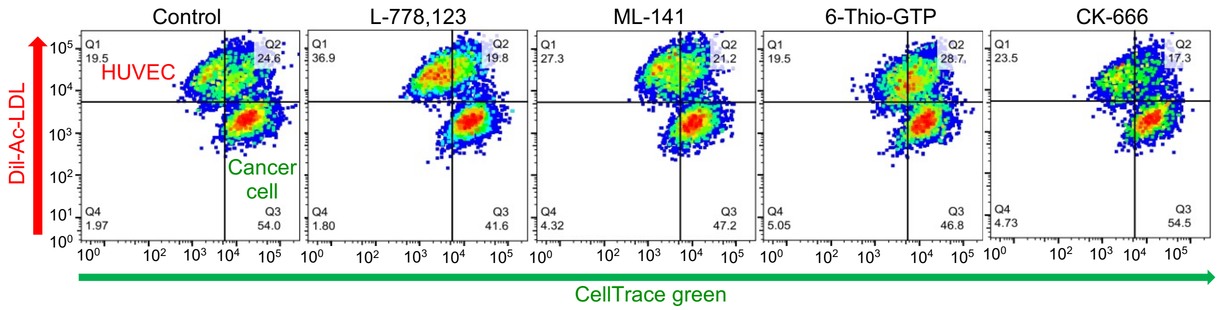

Supplementary Figure S9: Representative FACS plots show the reduction of intercellular CFSE transfer in the presence of inhibitors (each inhibitor concentration 10 µM and CK-666 40 µM). MDA-MB-231 cancer cells stained with CellTrace green (CFSE), and endothelial cells stained with Dil-Ac-LDL were plotted against both the fluorescence channels, respectively. Then, the transfer of cytoplasmic components CFSE from cancer cells to endothelial cells was checked. The population showing both red and green fluorescence, represents the population of endothelial cells accepted CFSE from cancer cells.

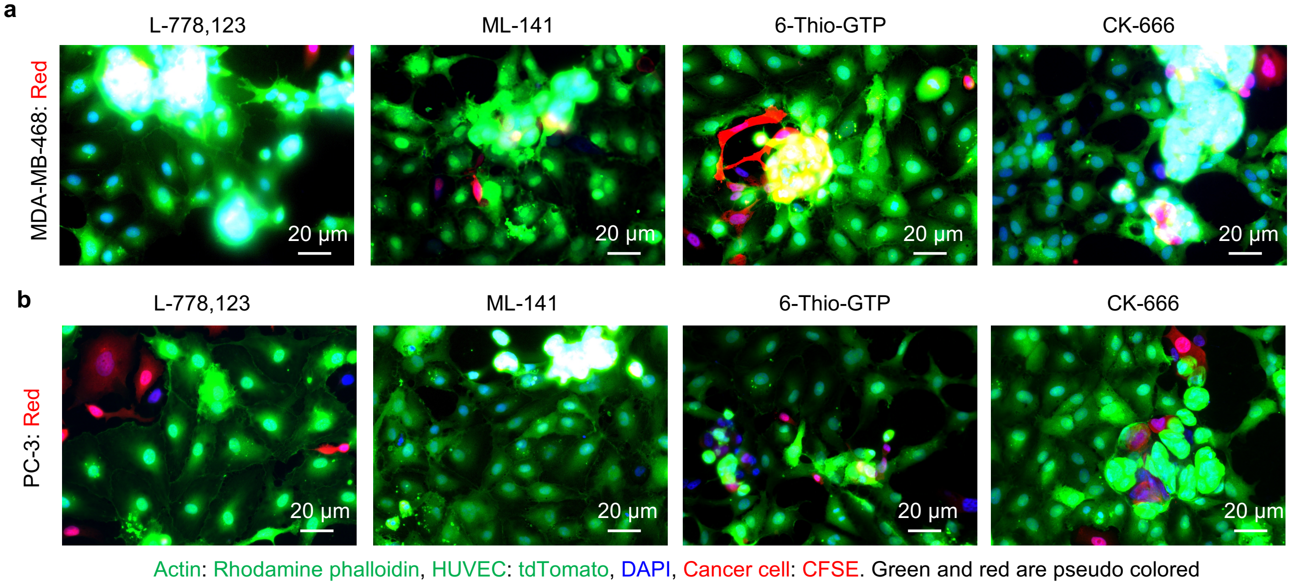

Supplementary Figure S10: (**a, b**) Representative fluorescence microscopy images show the effect of inhibitors in nanoscale communication between cancer cells (MDA-MB-468 and PC-3 cells) and the preformed endothelium in the 3D tumor matrix. Cancer cells were loaded with CellTrace green or CFSE and co-cultured with tdTomato labeled endothelial cells in the tumor matrix, in presence and absence of inhibitors. Each inhibitor (L-778,123, ML-141, 6-Thio-GTP and CK-666) was used separately at a concentration of 10 μM (40 μM for CK-666). A visible difference in the number of nanoscale communications and the reversion in shape of the cancer cells to 3D cellular aggregates or mammosphere-like structures were observed in presence of inhibitors. The co-culture was stained with rhodamine phalloidin to visualize the nanotubes. The images are pseudo colored to have a consistent color coding in all images throughout the manuscript. In the images, the green signal is from the phalloidin staining (which stain both cancer cells and endothelial cells), and red signal is from cancer cells. The yellow aggregates are because of merge of green and red signal in case of cancer cells.

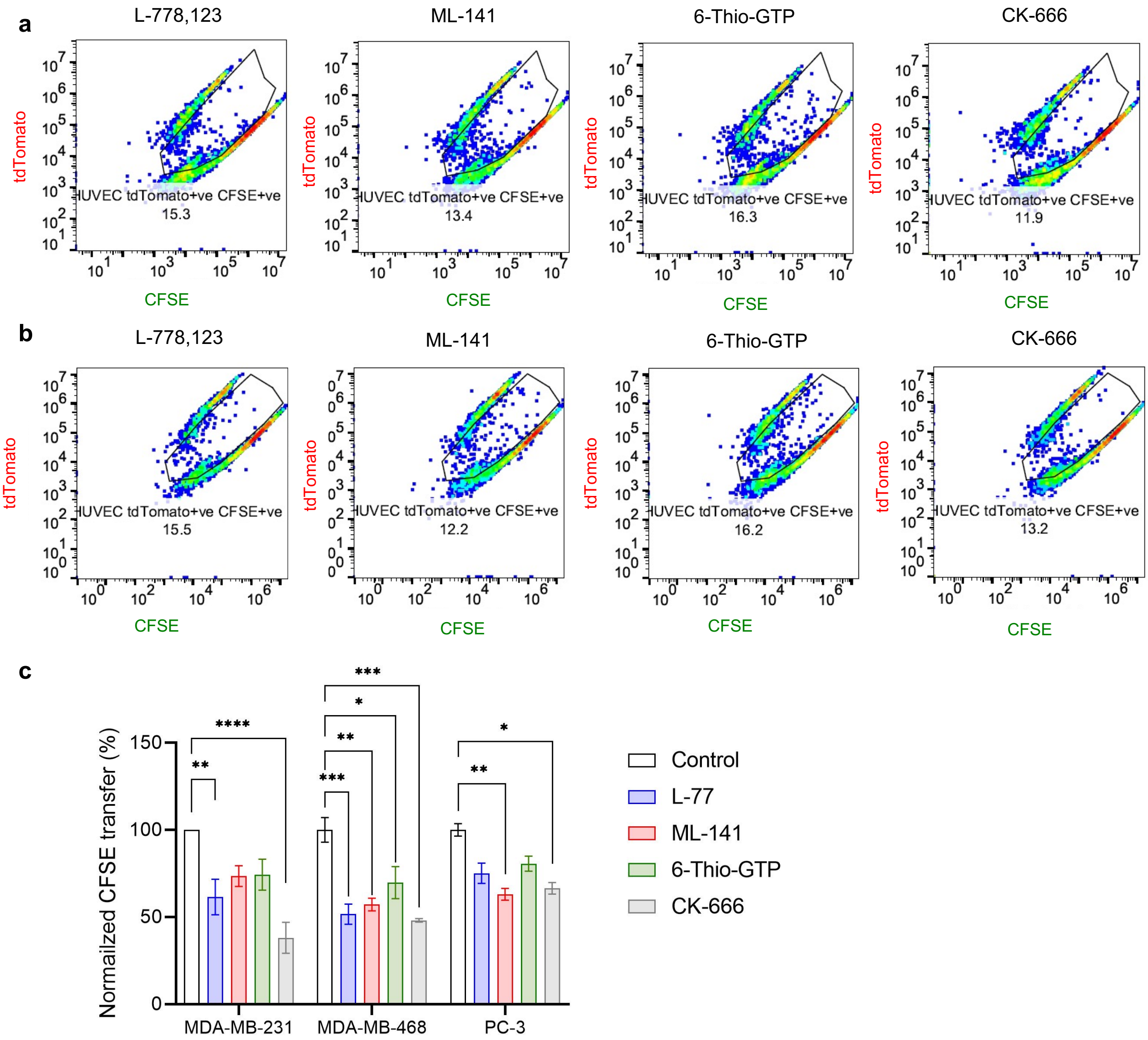

Supplementary Figure S11: Representative FACS plots show the reduction of intercellular CFSE transfer in the presence of inhibitors (each inhibitor concentration 10 µM and CK-666 40 µM). MDA-MB-468 (**a**) and PC-3 (**b**) cells were stained with CellTrace green (CFSE) and co-cultured with tdTomato labeled endothelial cells. The population showing both red and green fluorescence, represents the population of endothelial cells accepted CFSE from cancer cells. (**c**) Bar plot showing normalized CFSE transfer from cancer cells (MDA-MB-231, MDA-MB-468 and PC-3) to endothelial cells (HUVECs). The amount of HUVECs that have acquired CFSE from the cancer cells (% of cells that are both tdTomato +ve and CFSE +ve) has been obtained. The data in each inhibitor treatment condition was normalized according to the amount of CFSE transfer in case of control condition. Data represent mean ± SEM of at least three independent replicate (n>3). The statistical analysis was performed according to two-way ANOVA following Tukey's multiple comparison method and P values were presented as *P<0.05, **P<0.01, ***P<0.001, ****P<0.0001.

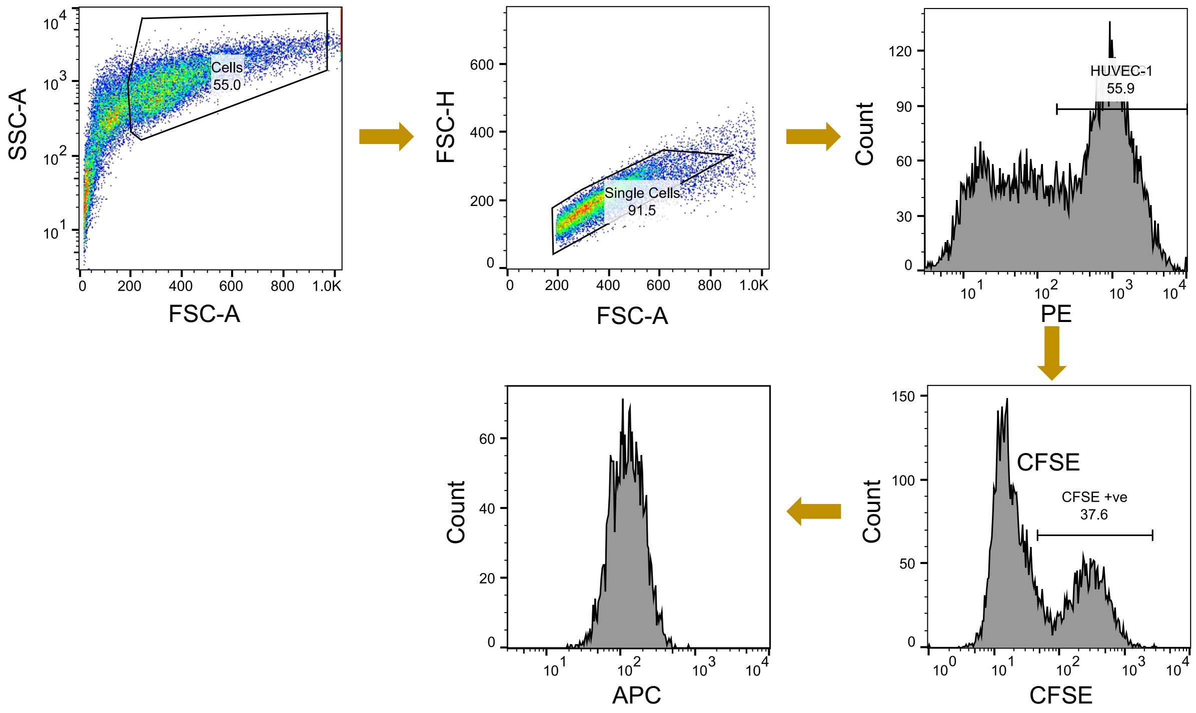

Supplementary Figure S12: The representative gating strategy of flow cytometric analysis to monitor the CD137 expression in HUVECs. MDA-MB-231 cancer cells (CFSE) were co-cultured with HUVECs (Dil-Ac-LDL) for 24 h. Endothelial cells were selected by Dil-Ac-LDL +ve gating. The CFSE +ve endothelial cells represent the endothelial cells accepted CFSE from cancer cells. Expression of CD137 (APC) was evaluated in the endothelial cells which has accepted CFSE from cancer cells (Dil-AC-LDL +ve and CFSE +ve cells).
